# Construction and Validation of a Nomogram Based on Radiomics and Clinical Features for Discerning Malignant Soft Tissue Tumors

**DOI:** 10.2174/0115734056409620251212101514

**Published:** 2026-01-15

**Authors:** Yusen Zhang, Chenyang Zhao, Licong Dong, Yun Tian, Heng Lv, Wangjie Wu, Lu Xie, Desheng Sun, Nan Zhuang, Haiqin Xie

**Affiliations:** 1 Department of Ultrasonography, Peking University Shenzhen Hospital, Shenzhen, China

**Keywords:** Ultrasonography, Soft tissue tumor, Radiomics, Traditional handcrafted imaging, Metastasis, STT diagnosis

## Abstract

**Objective:**

This study aimed to extract radiomic features from ultrasound (US) images of soft tissue tumors (STTs) and develop a diagnostic model for STTs using radiomic and clinical patient data.

**Methods:**

Three hundred and sixty-nine patients were recruited as the training group, with 249 benign and 120 malignant STTs, and 127 patients as the validation group, with 93 benign and 34 malignant STTs. We extracted the radiomic features of the US images using an open-source Python package. We selected the most relevant features using the least absolute shrinkage and selection operator (LASSO) regression. Then we used a combination of clinical indexes, radiomic features, and color-Doppler US to construct a diagnostic model for STTs. The diagnostic performance of the model was evaluated by measuring its sensitivity, specificity, area under the receiver operating curve (AUC), and calibration.

**Results:**

We selected 20 radiomic features of the US images. The model based on the clinical indexes, radiomic features, and color-Doppler scores showed good diagnostic performances on both the training [AUC: 0.97 (0.95-0.98)] and validation datasets [AUC: 0.93 (0.86-0.99)]. The model also presented good calibration with the original results.

**Discussion:**

We extracted radiomic features of ultrasound images of patients with STTs and constructed a clinical-imaging model for differentiating malignant and benign STT lesions. A nomogram displayed a clinical-imaging model, which showed good diagnostic efficacy and calibration in both the training and validation datasets. The clinical-imaging model has potential value for clinical use.

**Conclusion:**

The diagnostic model based on clinical, US radiomic, and imaging features presented a high diagnostic performance in STTs, which can have potential value in further clinical utilization.

## 1 INTRODUCTION

Soft tissue tumors (STTs) are benign or malignant neoplasms arising from soft tissues throughout the body, exhibiting diverse pathological types [1]. Despite their rarity compared with other common malignancies, STTs are health-threatening diseases that should not be ignored. In the early phase of malignant STTs, the symptoms are mild and can be easily misdiagnosed [2, 3]. Treating malignant STTs becomes difficult when they spread to adjacent tissues or metastasize. The metastasis and occurrence rates of soft tissue sarcomas are over 39% [4]. Moreover, the complexity of the clinical and pathological manifestations of STTs also hampers the diagnosis and treatment of the disease [5]. Therefore, more accurate and effective diagnostic tools are in demand to help clinicians tailor treatment strategies for these patients.

Ultrasound (US) is one of the most common imaging tools to diagnose STTs. Due to its safety and convenience, ultrasound has enjoyed widespread popularity for scanning patients with STTs clinically [6, 7]. However, there are still several drawbacks in STT diagnosis that need improvement. Firstly, the complex imaging manifestations of STTs make the diagnosis with US challenging. Moreover, operator dependence and intra-reader variance in the US hinder its accuracy. It is therefore vital to provide clinicians with a reliable and accurate diagnostic tool for STTs through US imaging.

Radiomics is a newly developed technique for medical imaging analysis that extracts high-throughput features from medical images. Unlike traditional handcrafted imaging features, radiomic features represent the spatial distributions of pixels or voxels that are not readily visible to the human eye. The extracted features could then be used to develop statistical diagnostic models for further clinical utility [8, 9]. The model based on radiomic features of ultrasonography has been developed for diagnosing cancers and predicting the prognosis of malignancies, such as breast cancer, hepatocellular carcinoma, and thyroid cancer [10-14].

We speculate that radiomics is a useful method for analyzing ultrasound images of STTs and aiding in diagnosis. To our knowledge, no prior studies have performed radiomic analysis of US imaging for STT lesions. Thus, in this study, we aimed to investigate the value of ultrasonic radiomics for differentiating malignant STTs and develop a diagnostic model for STTs based on radiomic features and clinical indicators.

## 2 MATERIALS AND METHODS

### 2.1 Ethics Approval

The study received approval from the hospital's ethics committee (approval number: 202200901) before it began. Since no interventional method was performed in this retrospective study, the informed consent was waived, as also approved by the ethics committee.

### 2.2 Patient Enrollment

This study was designed as a single-center, retrospective, and observational study. We retrospectively reviewed the picture archiving and communication system and medical record system of our hospital from July 2013 to December 2021. The patients diagnosed with STTs by pathological examinations after surgeries and biopsies were enrolled consecutively in this study. Tumor classification as benign or malignant was based on WHO criteria, but specific histopathological subtypes were not analyzed due to data limitations. The inclusion criteria included the following: (1) patients with complete records of clinical information and US imaging; (2) patients who received US scanning of the lesion within one month before surgery or biopsy (the one-month time frame for US scanning was selected to ensure imaging reflected the lesion’s state at pathological diagnosis, minimizing variability due to rapid tumor growth). The exclusion criteria included the following: (1) lesions originating from superficial organs, including the thyroid gland, breast, lymph nodes, and other exocrine glands (due to their distinct biological and radiomic characteristics compared to deep STTs); (2) STTs in the chest wall with a history of breast cancer were excluded if clinical judgment, based on history or imaging features (*e.g*., irregular margins, rapid growth), suggested metastasis, regardless of subsequent pathology.

### 2.3 Imaging Data Acquisition and Region of Interest (ROI) Delineation

The US examinations for the patients were performed *via* four different US machines (Philips IU22, Mindray Resona70, Aplio 500, and GE Healthcare EPIQ7) with high-resolution linear probes (frequency of 5-15MHz). The color-Doppler US settings for STT scanning included a pulse repetition frequency of 500-1000 Hz, a scale of 3-5 cm/s, a wall filter of 50-100 Hz, and a non-angled rectangular sampling box. Images of the longitudinal sections showing the lesions' largest diameters were recorded for radiomic analysis. Lesions larger than 10 cm were excluded, so the lesions could not be properly presented in a single plane. The US images used for radiomic analysis were not subjected to imaging optimization preprocessing. Ultrasound images were not preprocessed (*e.g*., intensity normalization or noise reduction) to preserve the original signal fidelity and capture subtle radiomic features reflective of tumor heterogeneity.

Two radiologists with 5 years of experience in musculoskeletal US manually outlined the ROIs of the selected STT images. A senior radiologist with 12 years of experience in musculoskeletal US checked the ROI delineation of all the selected images and modified the inappropriate ROIs. ROI segmentation was conducted using ITK-SNAP [15]. The three radiologists were blind to the recruited patients' clinical information and pathological results. The two radiologists also evaluated the color Doppler US images of the lesions and had them rechecked by the third senior radiologist. The detailed definitions of the four-grade blood flow grade are listed as follows: Grade 0, no blood flow; grade 1, one to two punctate or thin rod-shaped vessels within the lesion; grade 2, three to four punctate vessels or a penetrating vessel exceeding the radius of the lesion; grade 3, more than five punctate vessels or two penetrating vessels.

### 2.4 Radiomic Feature Extraction

The radiomic features of the US images were extracted by an open-source Python package (PyRadiomics) [16]. The extracted radiomic features included 13 features of two-dimensional shape-based statistics, 18 features of first-order statistics, 24 features of gray level cooccurrence matrix (GLCM), 14 features of gray level dependence matrix (GLDM), 16 features of gray level size zone matrix (GLSZM), and five features of neighboring gray tone difference matrix (NGTDM). First-order statistics describe the distribution of voxel intensities within the image region defined by the mask using commonly applied basic metrics. Two-dimensional shape-based statistics characterize the size and geometric properties of the ROI in two dimensions. GLCM describes the second-order joint probability function of an image region constrained by the mask. GLSZM quantifies gray level zones in an image. GLRLM quantifies gray level runs, defined as the length in the number of pixels of consecutive pixels with the same gray level value. NGTDM quantifies the difference between a gray value and the average gray value of its neighbors within distance ϭ. GLDM quantifies gray-level dependencies in an image. The feature pool was limited to 97 first-order, shape-based, and texture-based features to ensure interpretability and avoid overfitting, given the dataset size. Higher-order features (*e.g*., wavelet transforms) were excluded to maintain computational feasibility and model generalizability.

### 2.5 Radiomic Feature Reproducibility Assessment

To assess the reproducibility of radiomic features, 30 cases were randomly selected from the dataset. Two radiologists independently performed ROI segmentation, and after a 4-week interval, one radiologist repeated the segmentation for the same cases. The intraclass correlation coefficient (ICC) was calculated for all 97 radiomic features using the irr package in R (two-way mixed effects model, absolute agreement). Only features with ICC ≥ 0.75 were retained for subsequent model development.

### 2.6 Clinical Indexes Acquisition

Another two doctors recorded patients' clinical information with STTs from their electronic medical records. The recorded clinical indexes included sex (female or male), age, malignant history (yes or no), surgical history (yes or no), duration of the lesion, the growing speed of the lesion (slow: nearly no size change in one month; fast: significant size change in one month, more than 1 cm in diameter), pain in the lesion site (yes or no), tumor texture (soft: easily change in form after pressing; hard: do not deform after pressing), mobility (movable or immovable), skin changes in lesion site (yes or no), and location of the lesion (head and neck-1, trunk-2, upper extremity-3, or lower extremity-4).

### 2.7 Statistical Analysis

We constructed the model by using R (http://www.R-project.org) and EmpowerStats software (X&Y Solutions). About 75% of enrolled patients with STTs constituted the training cohort and the rest 25% were employed as the validation cohort. Firstly, we employed logistic regres-sion with the least absolute shrinkage and selection operator (LASSO) for dimensionality reduction to screen out the radiomics features that could contribute to the prediction model from the 97 radiomic features extracted by PyRadiomics. For LASSO analysis, the optimal parameter λ was acquired *via* 10-fold cross-validation. The coefficients of the features were calculated based on λ. The features with non-zero coefficients were selected. The study followed standard radiomic practices for feature extraction and model construction, though a specific radiomics checklist (*e.g*., CLEAR) was not used.

The selected radiomic features and collected clinical indexes were then used to build the diagnostic model. To avoid multicollinearity, the variables with a variance inflation factor value of less than 10 were included for model construction. The diagnostic model was built based on multivariate regression analysis. We used a backward stepwise variable selection method based on the Akaike information criterion (AIC) for model building. The model was internally validated using a bootstrap resampling method with 1,000 resamples.

To assess the incremental value of radiomic features, three models were constructed: (1) clinical features only, (2) radiomic features only, and (3) combined clinical + radiomic features. All models were trained and validated using the same dataset split and parameters. The performance metrics, including AUC, 95% CI, sensitivity, specificity, PPV, NPV, and accuracy, were calculated for each model. DeLong's test was used to compare AUCs between models. The integrated discrimination improvement (IDI) and net reclassification improvement (NRI) were calculated to quantify the incremental value of adding radiomic features to the clinical model.

Student's t-tests were used to compare categorical data, and Pearson chi-square tests were used to compare continuous data. We measured sensitivity, specificity, positive predictive value (PPV), negative predictive value (NPV), the receiver operating characteristic (ROC) curve, and the area under the ROC curve (AUC) to evaluate the diagnostic performance of the model. The final diagnostic model was represented as a nomogram. *P* < 0.05 was considered statistically significant.

## 3 RESULTS

### 3.1 Basic Clinical Characteristics of the Training and Validation Groups

A total of 369 patients were recruited as the training group, with 249 benign STTs and 120 malignant STTs, and 127 patients as the validation group, with 93 benign STTs and 34 malignant STTs. The mean age of the training and validation groups was 42.7 (32.8-53.9) and 41.6 (33.1-51.8) years old, respectively. Malignant history, pain, growth speed, texture, mobility, skin changes, and location significantly differed between malignant and benign STTs in the training dataset. Malignant history, speed, texture, mobility, and location showed significant differences between malignant and benign STTs in the validation dataset. The basic demographic and clinical characteristics of the training and validation groups are shown in Table **1**.

### 3.2 Radiomic Feature Extraction and Radiomic Score

A total of 97 radiomic features were extracted from the US images of the training dataset. They were reduced to 20 features for building the radiomic score by LASSO regression Fig. (**1A**, **B**). Table **2** summarizes the selected radiomic features and their detailed definitions. The radiomic score showed good performance in diagnosing STTs, with an AUC value of 0.813 for the training dataset Fig. (**1C**).

Of the 97 extracted radiomic features, 72 demonstrated good to excellent reproducibility (ICC ≥ 0.75). The model rebuilt with these stable features showed robust performance: AUC 0.96 (0.94-0.98) in the training set and 0.92 (0.85-0.99) in the validation set.

The formula of the radiomic score is shown as follows: Radiomic score = -0.00332*X1 + 0.0063*X2 + 0.00598*X3 - 0.0929*X4 + 0.00071*X5 - 0.00473*X6 - 0.00666* X7+ 0.03284*X8 + 2.03512*X9 + 68.12829*X10 + 0.79991*X11 + 1.23253*X12- 0.66551*X13 - 2.15822*X14 + 0.04019*X15 - 1e^-5^*X16 - 0.01455*X17 - 2.28252*X18 - 3.57946*X19 - 0.09685*X20.

### 3.3 Construction and Validation of the STT Diagnostic Model based on the Radiomic Score and Clinical Indexes

The variance inflation factors (VIFs) of the variables were first calculated to exclude multicollinearity, which ranged from 1 to 1.5, suggesting their availability for model construction (Table **S1**). We built the diagnostic model for STT using the radiomic score, the blood flow grade of Doppler US, and the clinical indexes of the patients, including age, malignant history, growing speed, texture, skin change, and location.

The formula of the clinical-imaging model using stepwise regression from Bootstrap is listed as follows:

Linear predictor = -97.28973 -1.41275*(sex=2) +0.06638*age +3.81824*(malignant history=1) -0.01546*dura- tion+2.52822*(speed=1) +1.71445*(location=2) -0.25197*(lo- cation=3) +1.98952*(location=4) +1.82800*(blood flow=1) +4.57183*(blood flow=2) +3.26284*(blood flow=3) +1.41752*radiomic score

Overall, the clinical imaging model showed an excellent diagnostic performance in differentiating malignant and benign STTs, with an AUC value of 0.97 (0.95-0.98) in the training dataset and 0.93 (0.86-0.99) in the validation dataset, as shown in Fig. (**2**). The clinical imaging model also showed good sensitivity in discerning malignant lesions in the two datasets [sensitivity in the training dataset: 0.93 (0.86-0.97), sensitivity in the validation dataset: 0.88 (0.73-0.97)]. The specificity of the model was also good in the two datasets [specificity in the training dataset: 0.91 (0.86-0.94), specificity in the validation dataset: 0.89 (0.81-0.95)]. The evaluation of the diagnostic performance of the model in the training and validation datasets is demonstrated in Table **3**. Due to the study’s focus on a combined clinical imaging model, separate ROC metrics for radiomic or clinical models alone were not evaluated.

To assess the incremental value of radiomic features, three models were constructed and compared. The combined model achieved the highest AUC in both cohorts, significantly outperforming the clinical and radiomic models alone (*p* < 0.001). IDI = 0.12 (*p* < 0.001) and NRI = 0.34 (*p* < 0.001) indicated significant improvement with the addition of radiomic features. Table **4** summarizes the performance metrics for all models in both training and validation sets.

**Fig. (3):**
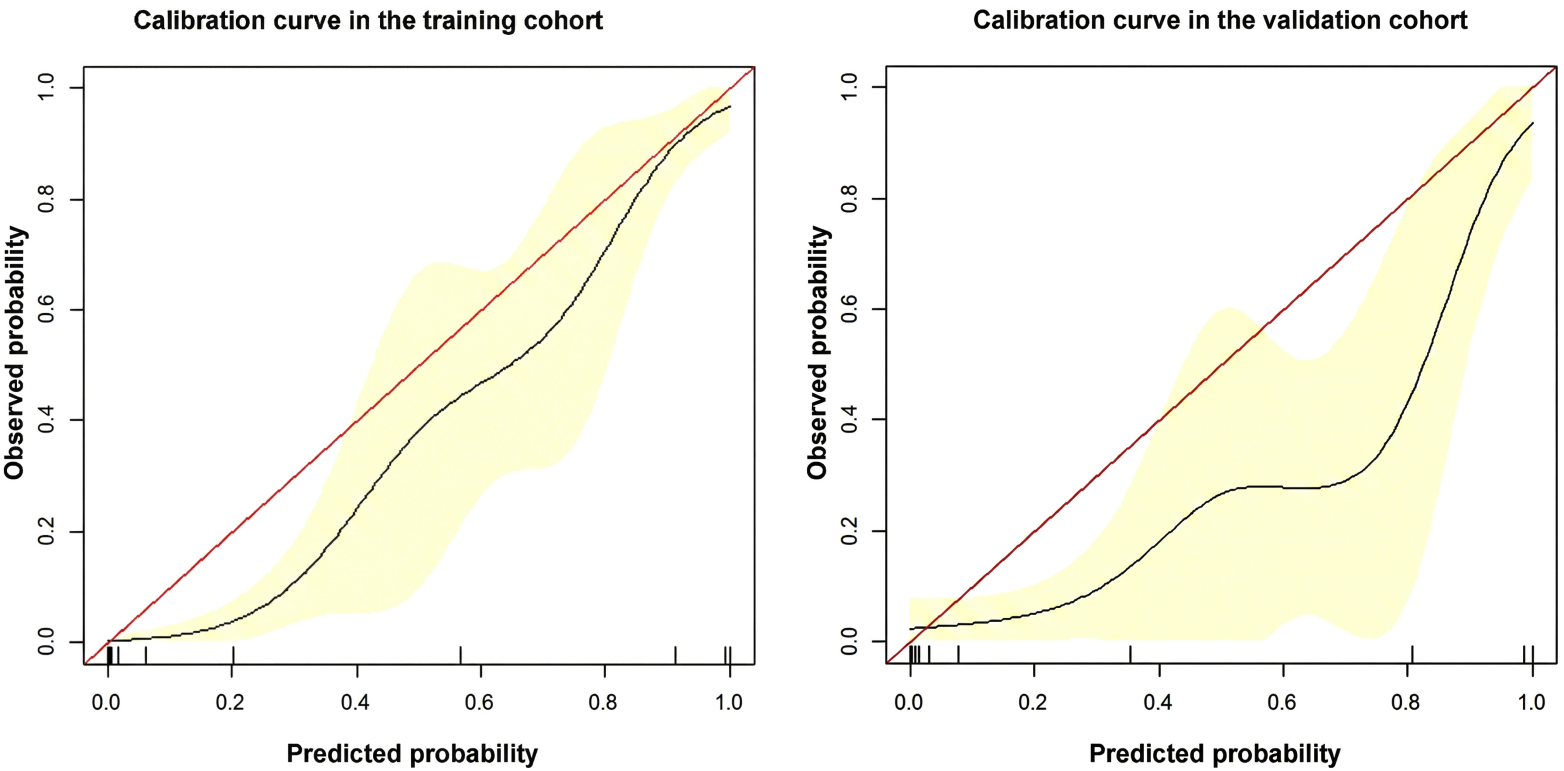
Calibration curves of the clinical imaging model in the training and validation datasets. Calibration curves for the model on the training and validation datasets are close to the diagonal line, indicating good agreement between observations and predictions.

Figure **3** shows the calibration curves of the model in the training and validation datasets to illustrate the consistency between the predicted malignant probability and actual results. The two cohorts' calibration curves are close to the diagonal line, indicating good agreement between observations and predictions.

### 3.4 Nomogram of the Clinical Imaging Model

The clinical imaging model was depicted as a nomogram for convenient clinical use. Examples of using the model to diagnose malignant and benign STT lesions are shown in Figs. (**4** and **5**).

## 4 DISCUSSION

In this study, we constructed a diagnostic model for differentiating malignant and benign STT lesions using radiomic features of US images and clinical indexes of the patients. A total of 369 patients were recruited as the training group, with 249 benign STTs and 120 malignant STTs, and 127 patients as the validation group, with 93 benign STTs and 34 malignant STTs. The model showed good diagnostic performances in the training and validation datasets, with an AUC value of 0.97 (0.95-0.98) in the training dataset and 0.93 (0.86-0.99) in the validation dataset. The model also presented good calibration with the original results. The model was further presented in the form of a nomogram for clinical utilization.

Surgery or biopsy is often recommended for patients with STTs for a precise diagnosis, which may lead to unnecessary biopsy for patients with low malignant risk and biopsy-related complications [1, 17, 18]. Therefore, clinicians have also attached importance to the imaging diagnosis of STTs. Due to the heterogeneous clinical and imaging characteristics of STTs, the accurate evaluation of the disease before surgery or biopsy is rather difficult. High-frequency US has been regarded as one of the first-line imaging methods for STTs due to its high resolution for superficial soft tissues, especially for those with small sizes [19, 20]. Earlier studies have employed US features, such as tumor margin, tumor location, tumor size, and vessel distributions, to discern malignant STTs, of which the accuracy was relatively low and unstable, ranging from 69% to 93% [21-24].

Consequently, the accuracy of US needs to be enhanced for better clinical employment. Researchers have built diagnostic models integrating US features to differentiate benign and malignant STTs. Chen et al. developed a semi-automated computer-aided diagnosis (CAD) system for STT diagnosis, which used five geometric features and 11 morphologic features of US images. The CAD system achieved an accuracy of 89% in 107 patients [25]. In the study by Wu et al., a nomogram for diagnosing STT was established based on the handcrafted US features and basic clinical characteristics of 200 patients with STTs. The nomogram presented a relatively higher diagnostic efficacy in discerning malignant STT than the model using US features alone, with an AUC value of 0.90 in the training dataset [26].

Compared to previous studies, our study included more STT cases to establish and validate the model. Furthermore, the model employed in this study achieved an AUC value of 0.95, demonstrating its exceptional ability to distinguish between benign and malignant entities. The model exhibited excellent diagnostic performance in the training and validation datasets, underscoring its reliability for STT diagnosis.

Radiomics is a novel imaging technique that automatically extracts quantitative data from medical images using sophisticated algorithms. The collected data from medical images were converted into high-dimensional features by radiomics. These features helped capture intricate tumor characteristics that human eyes could not discern [9]. Besides CT and MRI, radiomics has also emerged as a promising approach to analyzing US images for diagnostic purposes. US imaging-based radiomics has been employed in diagnosing malignancies and predicting the prognosis of various diseases, such as breast cancer, thyroid carcinoma, and hepatic cancer [27, 28]. In this study, the diagnostic or predictive models based on radiomic features of US images showed relatively good performances, which have been attached to potential clinical values. To our knowledge, this is the first study using radiomics to analyze US images of STTs. We extracted a wide range of features from the US images of STTs, including geometrical features, lesion texture, and internal echogenicity distributions. Computer algorithms automatically recognize these features, which are more objective in assessing the images than the conventional handcrafted features [29, 30].

The selected radiomic features achieved an AUC over 0.80 in discerning malignant STTs. To further enhance the diagnostic efficacy of the radiomic model, we incorporated clinical features into the model, including basic demographic features, clinical manifestations of tumors, skin changes, and tumor palpation. The comprehensive clinical imaging model exhibited high accuracy, sensitivity, and specificity, indicating its role in helping early detection of high-risk patients and the reduction of unnecessary biopsies simultaneously. Therefore, the clinical imaging model has the potential to serve as a valuable tool in clinical practice, facilitating more accurate and timely clinical decision-making.

The ICC analysis confirmed the reproducibility of our radiomic features, with 72 features showing ICC ≥ 0.75. Model comparisons demonstrated that radiomic features provided significant incremental value over clinical features alone, justifying their inclusion in the final model.

## 5 STUDY LIMITATION

Several limitations of this study need to be taken into consideration. First, we did not apply radiomic analysis for color-Doppler US images, assessed by a semi-quantitative grading method. Radiomic analysis for pseudo-color images may be used in future studies to incorporate color-Doppler radiomic features. Second, it is necessary to include an external cohort from other medical centers to further validate the clinical imaging model's availability. We should focus on prospective multicenter database collection in future investigations. Also, the addition of other modalities of ultrasound can allow for a more comprehensive diagnostic model. The absence of external validation thus limits the model’s generalizability. Future studies should include multicenter cohorts or public datasets to validate the model’s performance across diverse populations. Third, incorporating novel ultrasound techniques and conducting patient follow-up for prognosis prediction should be pursued to enhance the model's robustness and clinical applicability. Fourth, decision curve analysis was not performed due to data limitations. Future studies should incorporate DCA to quantify the clinical net benefit of the model across threshold probabilities. Fifth, the study did not analyze specific histopathological subtypes due to limited data, which may limit insights into subtype-specific radiomic patterns. Requiring complete records may introduce selection bias, as patients with incomplete data may reflect real-world variability. The inclusion of consecutive patients over eight years aimed to mitigate this bias. Finally, excluding cases with suspected metastasis may have omitted some non-metastatic STTs, potentially affecting the study’s completeness. Thus, excluding superficial organ-derived lesions limits the model’s applicability to all STTs, particularly those in superficial tissues, which may have different radiomic patterns.

In addition, the retrospective design limited the study’s ability to account for real-time clinical variability. Prospective studies are needed to validate the model in continuous patient populations. We acknowledge that the use of four different ultrasound systems may have introduced variability in radiomic feature extraction. While standardized imaging protocols were employed, additional calibration methods could further enhance consistency across platforms. Future studies should explore cross-platform standardization techniques. The study also did not calculate intraclass correlation coefficients (ICC) for radiomic features due to resource limitations. ROI delineation was performed by experienced radiologists to ensure consistency, but future studies should include ICC analysis to quantify feature stability.

## CONCLUSION

We extracted radiomic features of ultrasound images from patients with STTs. We constructed a clinical imaging model for differentiating malignant and benign STT lesions using the radiomic features of US images and clinical indexes of the patients. A nomogram displayed the clinical imaging model, which showed good diagnostic efficacy and calibration in both the training and validation datasets. The clinical imaging model can thus have potential value in clinical utilization.

## Figures and Tables

**Fig. (1):**
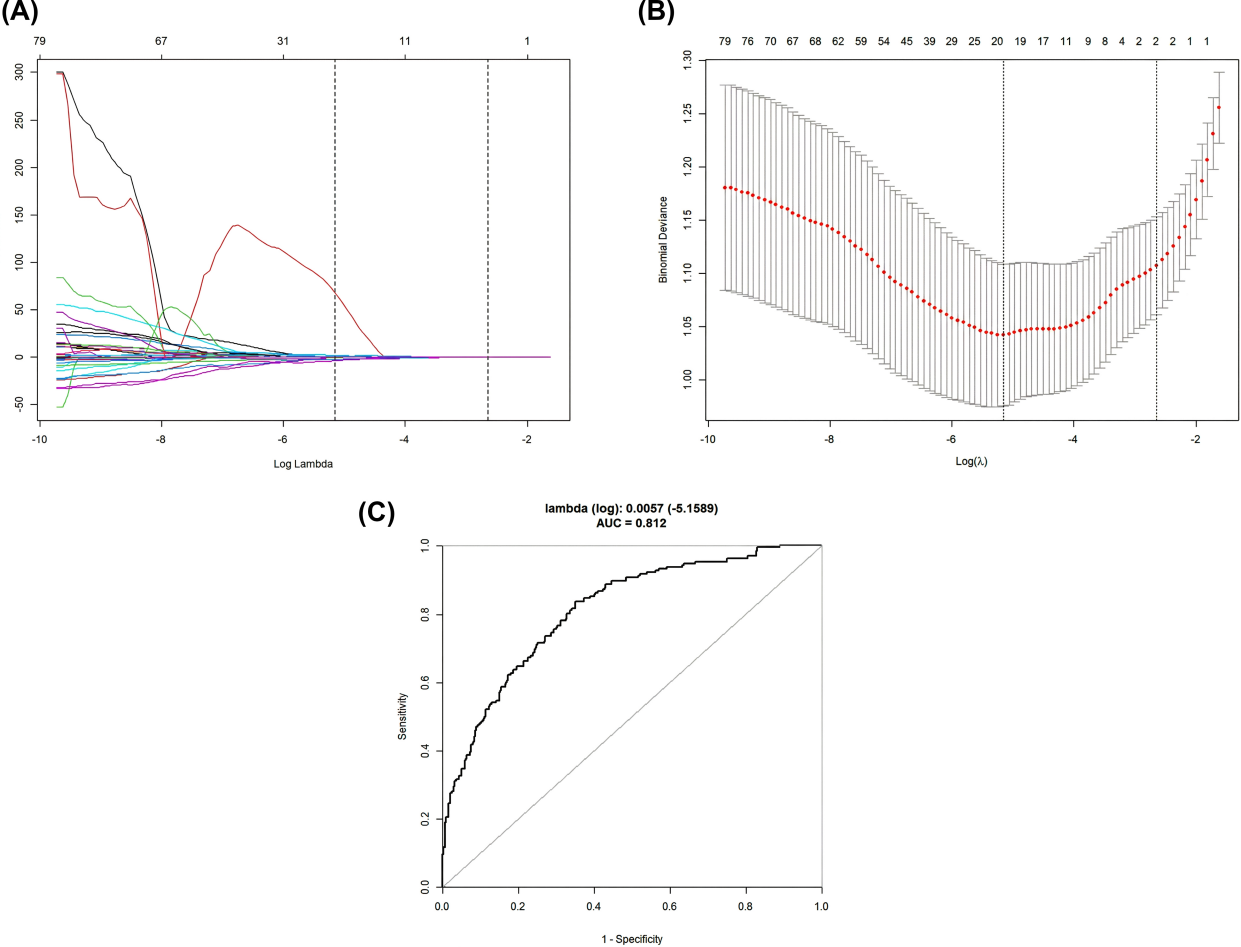
The LASSO regression of the radiomic features. (**A**) The tuning parameter λ was selected through 10-fold cross-validation. The feature selection was performed using the value of λ, resulting in the minimum average binomial deviance. The optimal values were marked with dotted vertical lines. (**B**) The LASSO coefficient profiles of the 90 radiomics features are displayed. The optimal outcome was achieved with 20 non-zero coefficients, as indicated by the vertical line. (**C**) The ROC curve of the selected features for STT diagnosis.

**Fig. (2):**
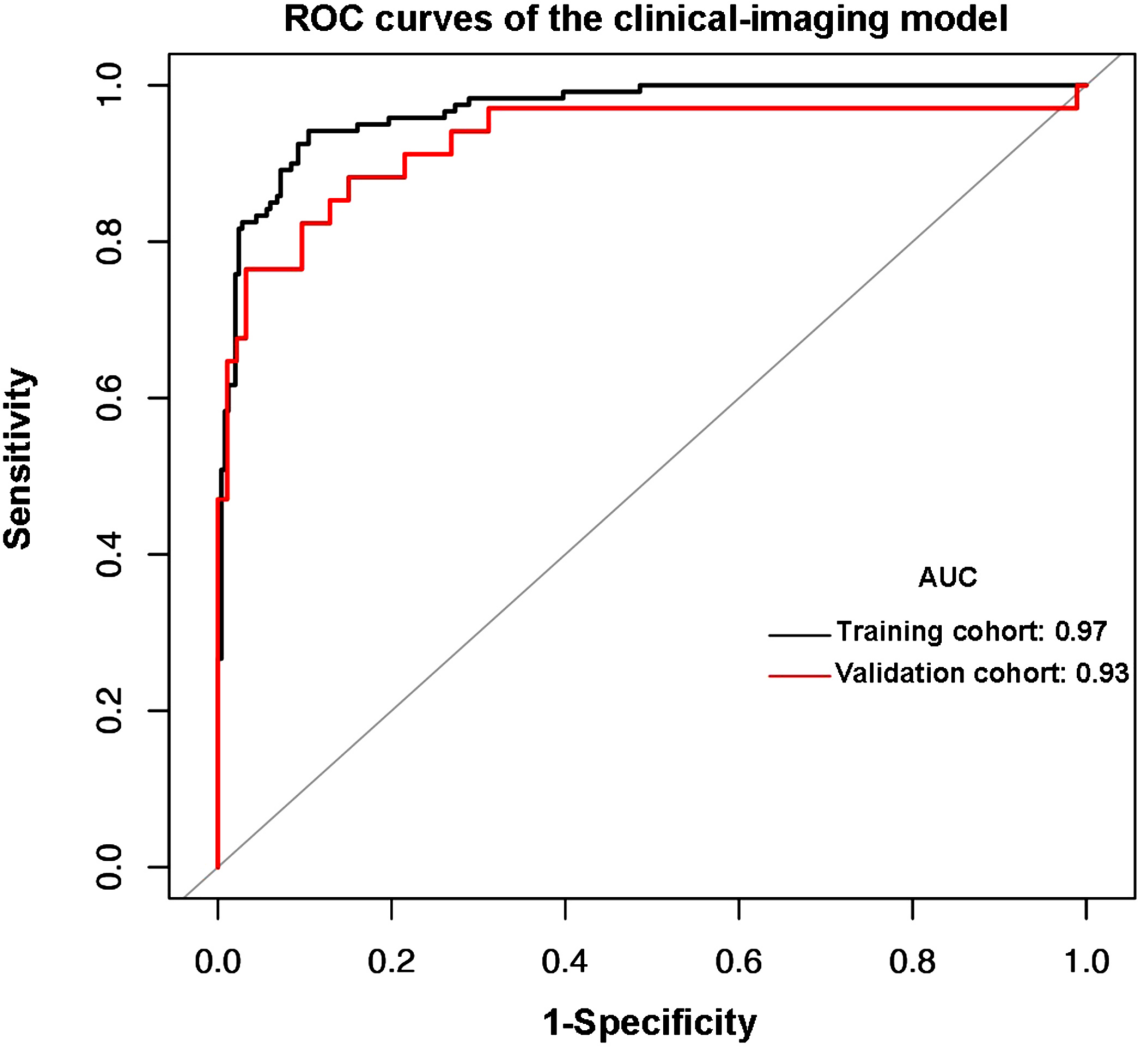
ROC curves of the clinical imaging model in the training and validation datasets. The clinical imaging model showed an excellent diagnostic performance in differentiating malignant and benign STTs, with an AUC value of 0.97 (0.95-0.98) in the training dataset and 0.93 (0.86-0.99) in the validation dataset.

**Fig. (4):**
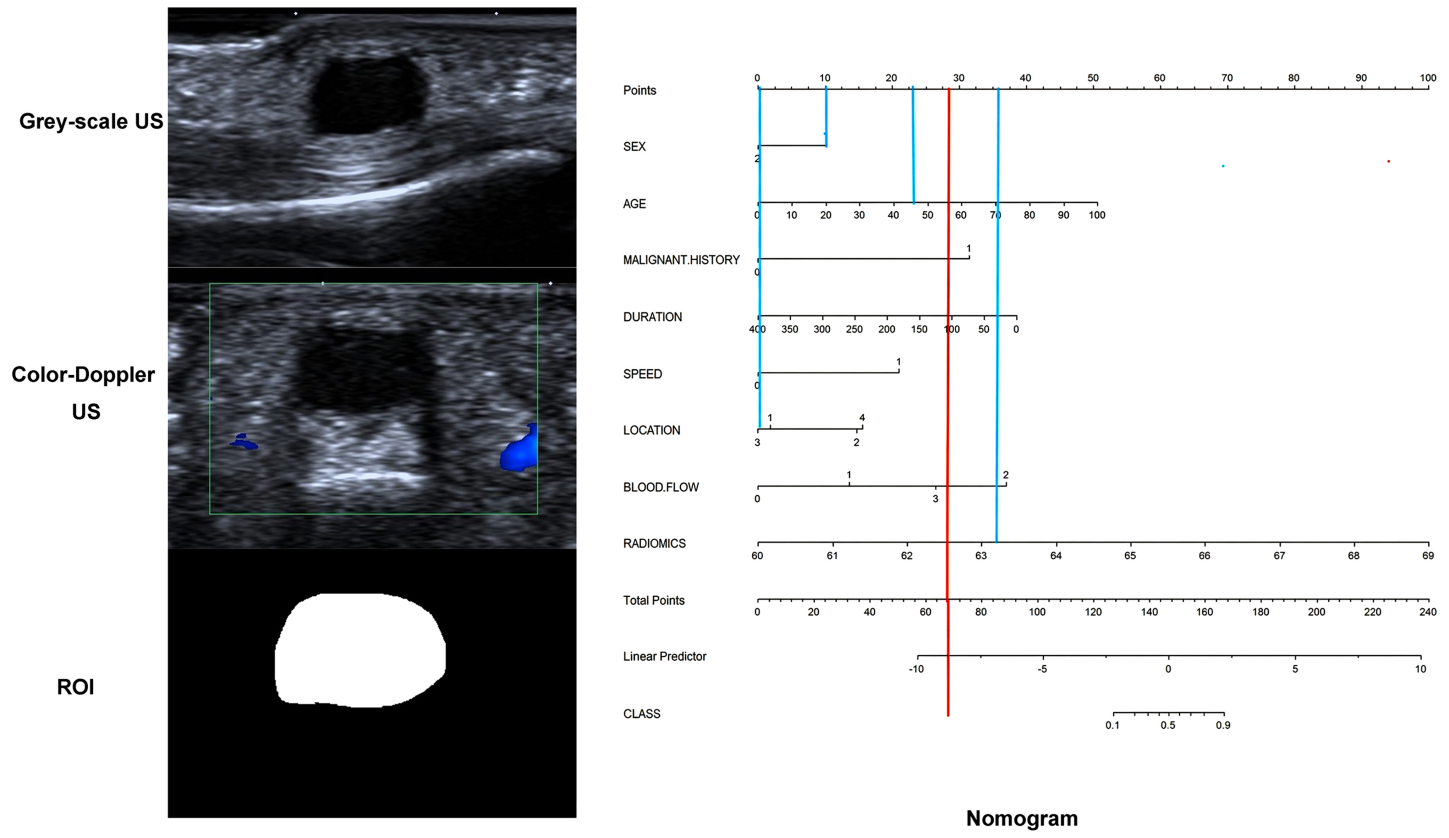
Example of the clinical imaging model diagnosing a benign STT lesion. A 44-year-old female diagnosed with STT on the right upper extremity received ultrasound scanning. The grey-scale US, color-Doppler US, and ROI segmentation of the grey-scale US are presented on the left side of the figure. The nomogram shows that the patient's total score was approximately 70, with a malignant risk of less than 0.1.

**Fig. (5):**
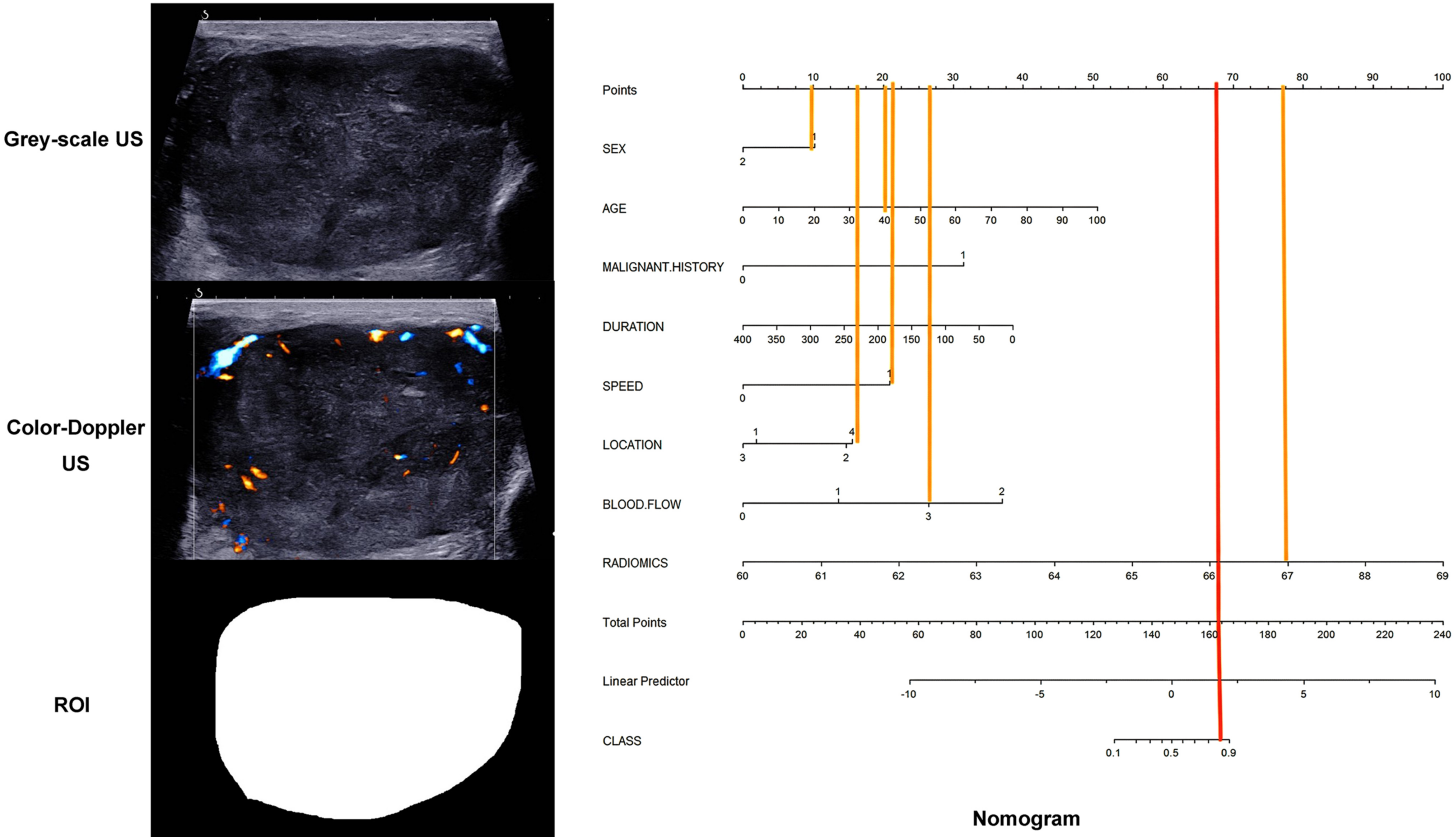
Example of the clinical imaging model diagnosing a malignant STT lesion. A 40-year-old male diagnosed with STT on the left lower extremity received ultrasound scanning. The lesion grew fast and had a blood flow grade of 3. The grey-scale US, color-Doppler US, and ROI segmentation of the grey-scale US are presented on the left side of the figure. The nomogram shows that the patient's total score was over 170, with a malignant risk ranging from 0.8 to 0.9.

**Table 1:** Basic information of the training and validation cohorts.

**Training Cohort**	**Benign**	**Malignant**	***P***	**Validation Cohort**	**Benign**	**Malignant**	***P***
**N**	249	120			93	34	
**Age**	37.00 (29.00-50.00)	54.50 (40.75-62.00)	<0.001		40.00 (33.00-51.00)	46.00 (33.25-54.00)	0.19
**Duration**	35.20 (55.39) 12.00 (4.00-48.00)	12.89 (25.18) 3.50 (1.00-10.00)	<0.001		31.66 (41.22) 12.00 (4.00-36.00)	19.63 (34.68) 6.00 (1.62-21.00)	0.011
**Sex**			<0.001				0.728
**Male**	119 (47.79%)	81 (67.50%)			47 (50.54%)	16 (47.06%)	
**Female**	130 (52.21%)	39 (32.50%)			46 (49.46%)	18 (52.94%)	
**Malignant history**			<0.001				<0.001
**No**	241 (96.79%)	72 (60.00%)			92 (98.92%)	18 (52.94%)	
**Yes**	8 (3.21%)	48 (40.00%)			1 (1.08%)	16 (47.06%)	
**Pain**			<0.001				0.187
**No**	200 (80.32%)	76 (63.33%)			78 (83.87%)	25 (73.53%)	
**Yes**	49 (19.68%)	44 (36.67%)			15 (16.13%)	9 (26.47%)	
**Speed**			<0.001				<0.001
**No**	237 (95.18%)	92 (76.67%)			89 (95.70%)	22 (64.71%)	
**Yes**	12 (4.82%)	28 (23.33%)			4 (4.30%)	12 (35.29%)	
**Texture**			<0.001				0.001
**Soft**	192 (77.11%)	48 (40.00%)			78 (83.87%)	19 (55.88%)	
**Hard**	57 (22.89%)	72 (60.00%)			15 (16.13%)	15 (44.12%)	
**Mobility**			<0.001				0.008
**Soft**	204 (81.93%)	68 (56.67%)			78 (83.87%)	21 (61.76%)	
**Hard**	45 (18.07%)	52 (43.33%)			15 (16.13%)	13 (38.24%)	
**Skin change**			0.004				0.087
**No**	245 (98.39%)	111 (92.50%)			91 (97.85%)	31 (91.18%)	
**Yes**	4 (1.61%)	9 (7.50%)			2 (2.15%)	3 (8.82%)	
**Location**			<0.001				0.047
**Head and neck**	54 (21.69%)	16 (13.33%)			26 (27.96%)	6 (17.65%)	
**Trunk**	63 (25.30%)	65 (54.17%)			22 (23.66%)	16 (47.06%)	
**Upper extremity**	88 (35.34%)	12 (10.00%)			28 (30.11%)	5 (14.71%)	
**Lower extremity**	44 (17.67%)	27 (22.50%)			17 (18.28%)	7 (20.59%)	

**Table 2:** The selected radiomic features for model construction and their definitions.

**Variable**	**Radiomic features**	**Definition**
X1	original_shape_Maximum2DDiameterColumn	The largest pairwise Euclidean distance between tumor surface mesh vertices
X2	original_shape_Maximum2DDiameterRow	The largest pairwise Euclidean distance between tumor surface mesh vertices
X3	original_shape_MinorAxisLength	The second-largest axis length of the ROI-enclosing ellipsoid
X4	original_shape_SurfaceVolumeRatio	Multiplying the number of pixels in the ROI by the surface area of a single pixel
X5	original_firstorder_Median	Median gray level intensity within the ROI
X6	original_firstorder_Skewness	The asymmetry of the distribution of values with respect to the mean value.
X7	original_glcm_ClusterShade	The skewness and uniformity of the GLCM. A higher cluster shade implies greater asymmetry with respect to the mean
X8	original_glcm_ClusterTendency	Groupings of voxels with similar gray-level values
X9	original_glcm_DifferenceEntropy	Randomness/variability in neighborhood intensity value differences
X10	original_glcm_Idmn (Inverse Difference Moment Normalized)	Local homogeneity of an image
X11	original_glcm_Imc1 (Informational Measure of Correlation)	Quantifying the complexity of the texture
X12	original_glcm_MaximumProbability	Occurrences of the most predominant pair of neighboring intensity values
X13	original_gldm_DependenceEntropy	The number of connected voxels within distance δ dependent on the center voxel
X14	original_gldm_DependenceVariance	The number of connected voxels within distance δ dependent on the center voxel
X15	original_gldm_GrayLevelVariance	The variance in gray level intensities for the zones
X16	original_glszm_LargeAreaHighGrayLevelEmphasis	The proportion in the image of the joint distribution of larger-sized zones with higher gray-level values
X17	original_glszm_LargeAreaLowGrayLevelEmphasis	The proportion in the image of the joint distribution of larger-sized zones with lower gray-level values
X18	original_glszm_SmallAreaLowGrayLevelEmphasis	The proportion in the image of the joint distribution of smaller-sized zones with lower gray-level values
X19	original_glszm_ZonePercentage	The coarseness of the texture determined by taking into account the ratio of the number of zones and the number of voxels in the ROI
X20	original_ngtdm_Busyness	The change from a pixel to its neighbor

**Table 3:** Diagnostic performance of the clinical imaging model in the training and validation datasets.

	**Training Dataset**	**Validation Dataset**
AUC	0.97 (0.95-0.98)	0.93 (0.86-0.99)
Specificity	0.91 (0.86-0.94)	0.89 (0.81-0.95)
Sensitivity	0.93 (0.86-0.97)	0.88 (0.73-0.97)
Accuracy	0.91 (0.88-0.94)	0.89 (0.82-0.94)
Positive likelihood ratio	10.0 (6.8-14.8)	8.2 (4.5-14.9)
Negative likelihood ratio	0.08 (0.04-0.16)	0.13 (0.05-0.33)
Positive predictive value	0.83 (0.77-0.88)	0.75 (0.62-0.85)
Negative predictive value	0.96 (0.93-0.98)	0.95 (0.89-0.98)

**Table 4:** Performance comparison of the three models.

**Model**	**Cohort**	**AUC (95% CI)**	**Sensitivity**	**Specificity**	**PPV**	**NPV**	**Accuracy**
Clinical	Training	0.88 (0.85-0.91)	0.85	0.82	0.74	0.91	0.83
	Validation	0.84 (0.76-0.92)	0.82	0.80	0.71	0.89	0.81
Radiomic	Training	0.89 (0.86-0.92)	0.86	0.83	0.75	0.92	0.84
	Validation	0.86 (0.78-0.94)	0.84	0.82	0.73	0.90	0.83
Combined	Training	0.97 (0.95-0.98)	0.93	0.91	0.83	0.96	0.91
	Validation	0.93 (0.86-0.99)	0.88	0.89	0.75	0.95	0.89

## Data Availability

The data of current study are available from corresponding authors, [N.Z] and [H.X], on a reasonable request.

## LIST OF ABBREVIATIONS

STTs: Soft tissue tumors
US: Ultrasound
GLCM: Gray level co-occurrence matrix
GLDM: Gray level dependence matrix
GLSZM: Gray level size zone matrix
NGTDM: Neighboring gray tone difference matrix
ICC: Intraclass correlation coefficient
LASSO: Least absolute shrinkage and selection operator
AIC: Akaike information criterion
IDI: Integrated discrimination improvement
NPV: Negative predictive value
PPV: Positive predictive value
ROC: Receiver operating characteristic

## STANDARDS OF REPORTING

STROBE guidelines were followed.
