# Supplementary material for: Construction and Validation of a Nomogram Based on Radiomics and Clinical Features for Discerning Malignant Soft Tissue Tumors
Source: Curr Med Imaging. 2026 Jan 15;22:e15734056409620. doi: 10.2174/0115734056409620251212101514 (PMC13445551; doi:10.2174/0115734056409620251212101514)
Supplement: Supplementary file 1 [file CMIM-22-E15734056409620_SD1.pdf]

1875-6603/26

Send Orders for Reprints to reprints@benthamscience.net

BENTHAM SCIENCE

CrossMark

Current Medical Imaging

Content list available at: https://benthamscience.com/journals/cmir

Supplementary Material

Construction and Validation of a Nomogram Based on Radiomics and Clinical Features for Discerning Malignant Soft Tissue Tumors

Yusen Zhang[1,*], Chenyang Zhao[1,*], Licong Dong[1,*], Yun Tian[1], Heng Lv[1,#], Wangjie Wu[1], Lu Xie[1], Desheng Sun[1], Nan Zhuang[1,#] and Haiqin Xie[1,#]

[1]*Department of Ultrasonography, Peking University Shenzhen Hospital, Shenzhen, China*

Table 1. Collinearity VIF selection of the variables.

	VIF
Sex	1
Age	1.1
Malignant history	1.1
Duration	1.1
Pain	1.1
Speed	1.2
Texture	1.5
Mobility	1.5
Skin change	1.1
Location	1.1
Radiomic score	1.2

© 2026 The Author(s). Published by Bentham Science Publisher.

cc BY

This is an open access article distributed under the terms of the Creative Commons Attribution 4.0 International Public License (CC-BY 4.0), a copy of which is available at: https://creativecommons.org/licenses/by/4.0/legalcode. This license permits unrestricted use, distribution, and reproduction in any medium, provided the original author and source are credited.

HOW TO CITE:
Zhang Y, Zhao C, Dong L, Tian Y, Lv H, Wu W, Xie L, Sun D, Zhuang N, Xie H. Construction and Validation of a Nomogram Based on Radiomics and Clinical Features for Discerning Malignant Soft Tissue Tumors. Curr Med Imaging, 2026; 22: e15734056409620.
http://dx.doi.org/10.2174/0115734056409620251212101514
